# Impact of suboptimal or inappropriate treatment on healthcare resource use and cost among patients with uncomplicated urinary tract infection: an analysis of integrated delivery network electronic health records

**DOI:** 10.1186/s13756-022-01170-3

**Published:** 2022-11-04

**Authors:** Jason Shafrin, Alen Marijam, Ashish V. Joshi, Fanny S. Mitrani-Gold, Katie Everson, Rifat Tuly, Peter Rosenquist, Michael Gillam, Maria Elena Ruiz

**Affiliations:** 1 PRECISIONheor, Los Angeles, CA USA; 2 GSK, Collegeville, PA USA; 3 PRECISIONheor, Austin, TX USA; 4 PRECISIONheor, Washington, DC, USA; 5 MedStar Health, Washington, DC, USA

**Keywords:** Urinary tract infection, Uncomplicated urinary tract infection, Antibiotic resistance, Cost, Healthcare resource use

## Abstract

**Background:**

Although uncomplicated urinary tract infections (uUTIs; occurring in female patients without urological abnormalities or history of urological procedures or complicating comorbidities) are one of the most common community infections in the United States (US), limited data are available concerning associations between antibiotic resistance, suboptimal prescribing, and the economic burden of uUTI. We examined the prevalence of suboptimal antibiotic prescribing and antibiotic resistance and its effects on healthcare resource use and costs.

**Methods:**

This retrospective cohort study utilized electronic health record data from a large Mid-Atlantic US integrated delivery network database, collected July 2016–March 2020. Female patients aged ≥ 12 years with a uUTI, who received ≥ 1 oral antibiotic treatment within ± 5 days of index uUTI diagnosis, and had ≥ 1 urine culture with antimicrobial susceptibility test, were eligible for inclusion in the study. The study examined the proportion of antibiotics that were inappropriately or suboptimally prescribed among patients with confirmed uUTI, and total healthcare costs (all-cause and UTI-related) within 6 months after a uUTI, stratified by antibiotic susceptibility and/or inappropriate or suboptimal treatment. Patient outcomes were assessed after 1:1 propensity score matching of patients with antibiotic-susceptible versus not-susceptible isolates and then by other covariates (e.g., demographics and recent healthcare use). A similar propensity score calculation was used to analyze the effect of inappropriate/suboptimal treatment on health outcomes. Costs were adjusted to 2020 US dollars ($).

**Results:**

Among 2565 patients with a uUTI included in the analysis, the most commonly prescribed antibiotics were nitrofurantoin (61%), trimethoprim-sulfamethoxazole (19%), and ciprofloxacin (15%). More than one-third of the sample (40.2%) had isolates that were not-susceptible to ≥ 1 antibiotic indicated for treating patients with uUTI. Two-thirds (66.6%) of study-eligible patients were prescribed appropriate treatment; 29.9% and 11.9% were prescribed suboptimal and/or inappropriate treatment, respectively. Inappropriate or suboptimally prescribed patients had greater all-cause and UTI-related costs compared with appropriately prescribed patients. Differences were most striking among patients with antibiotic not-susceptible isolates.

**Conclusions:**

These findings highlight how the increasing prevalence of antibiotic resistance combined with suboptimal treatment of patients with uUTI increases the burden on healthcare systems. The finding underlines the need for improved prescribing accuracy by better understanding regional resistance rates and developing improved diagnostic tests.

## Background

Uncomplicated urinary tract infections (uUTIs/acute cystitis) are one of the most common community infections in the United States (US) [1]. By definition, uUTIs occur in patients with no functional or anatomical urological abnormalities, and no history of recent urological procedures or complicating comorbidities [2, 3]. Approximately 30–40% of women report at least one uUTI in their lifetime, and the majority of these will be prescribed antibiotics for management [4]. Treatment guidelines [5–9] recommend several treatment options for uUTI, including antibiotic agents such as nitrofurantoin, trimethoprim-sulfamethoxazole (SXT), or fosfomycin. An optimal agent is selected on a case-by-case basis, depending on a number of different factors, and real-world prescription practices vary greatly [6]. Importantly, treatment guidelines cannot always be universally applied to all patients, for reasons such as allergies, intolerances, local resistance rates, and comorbidities [10, 11], which can result in the guideline-defined “inappropriate” prescribing of antibiotics.

While the annual costs associated with uUTI are estimated to be $1.6 billion in the US [12], antibiotic resistance in uUTIs provides an additional burden on the healthcare system [6]. Increased resistance to antibiotics and treatment failure rates result in a greater cost to treat patients with antibiotic-not-susceptible infections compared to patients with antibiotic-susceptible infections [13, 14]. In addition, there are limited current data available on the overall prevalence of inappropriate or suboptimal prescribing of antibiotics for the treatment of uUTI, and little information on what impact this has upon treatment costs. Studies to date suggest that the prevalence of inappropriate and/or suboptimal antibiotic prescribing is high [15–17].

This study used real-world data from US female outpatients with uUTI to assess the prevalence of inappropriate or suboptimal antibiotic prescribing (based on Infectious Diseases Society of America guidelines [7]), and the effects of inappropriate or suboptimal antibiotic prescribing on healthcare resource use (HCRU) and costs.

## Methods

### Study design

**Fig. 1:**
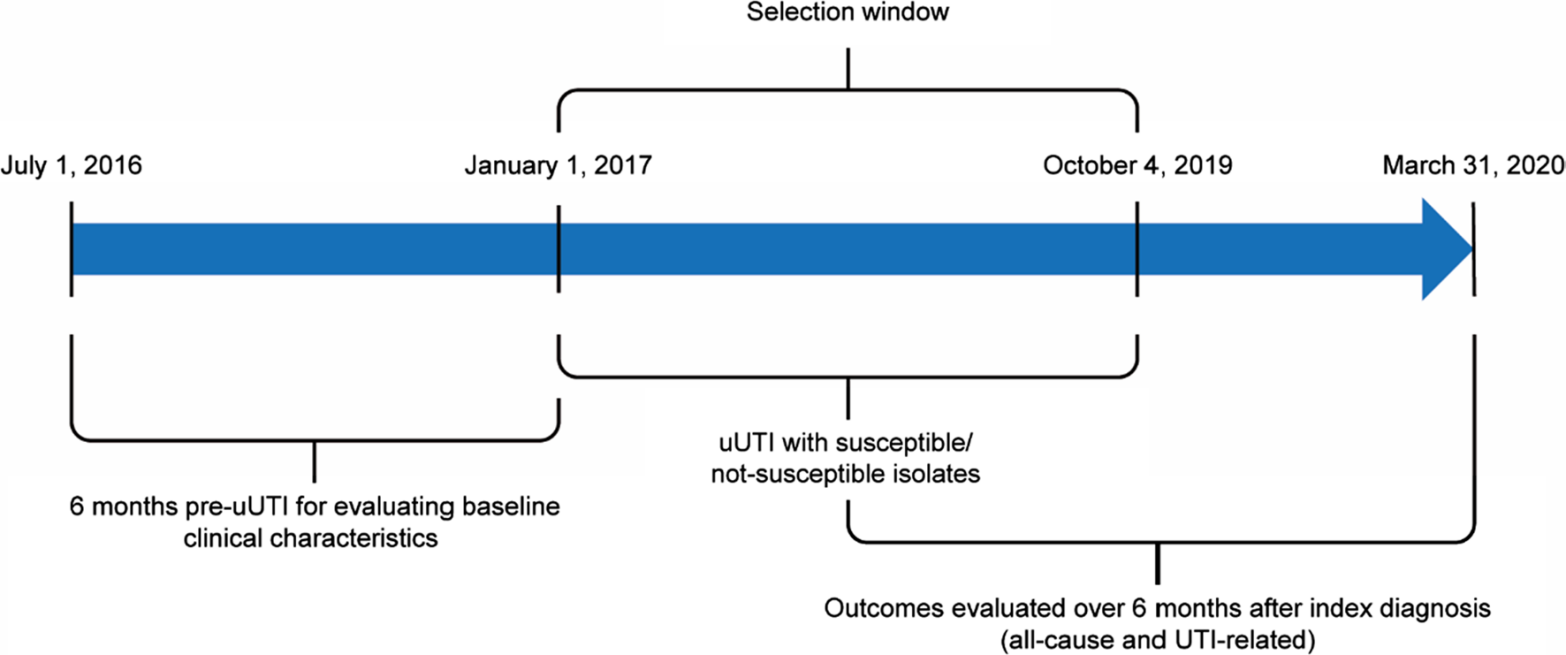
Overview of study design (patients with uUTI with or without antibiotic resistance) [29]. *UTI* uncomplicated urinary tract infection, *uUTI* uncomplicated urinary tract infection

This was a retrospective cohort study of electronic health record (EHR) data from a large Mid-Atlantic US integrated delivery network database that encompasses 10 hospitals and 300 inpatient, outpatient, and urgent care sites across 2 states. Data were collected between July 2016 and March 2020 (Fig. 1). The database relies on the Cerner PowerChart EHR platform and contains various information, including but not limited to data concerning patient diagnosis, prescriptions, procedures, and laboratory values. The index date was defined as the date of a patient’s first uUTI diagnosis or urine culture with available antimicrobial susceptibility test results.

This study complied with all applicable laws regarding subject privacy. No direct subject contact or primary collection of individual human subject data occurred, and since data were de-identified, informed consent and ethics committee approval were not required.

### Patients

Female patients aged ≥ 12 years at the time of index uUTI diagnosis were eligible for inclusion in the study. Patients were required to have had ≥ 1 oral antibiotic prescription within ± 5 days of the index uUTI diagnosis with the first prescription identified as the initial therapy, confirmed by ≥ 1 urine culture with antimicrobial susceptibility testing performed. In addition, patients had diagnosed primary or secondary uUTI (per International Classification of Disease [ICD], Ninth Revision [ICD-9] and/or Tenth Revision [ICD-10] codes), or had a urine culture with ≥ 10^4^ colony forming unit (CFU)/mL of a uropathogen. Patients with an ICD-9/10 diagnosis code for acute cystitis and UTI site not specified were included. In some cases, patients may have been diagnosed with having a uUTI based on consultation alone (UTI symptoms) and started initial antibiotic therapy ahead of confirmation of diagnosis via culture/antimicrobial susceptibility testing results.

**Fig. 2:**
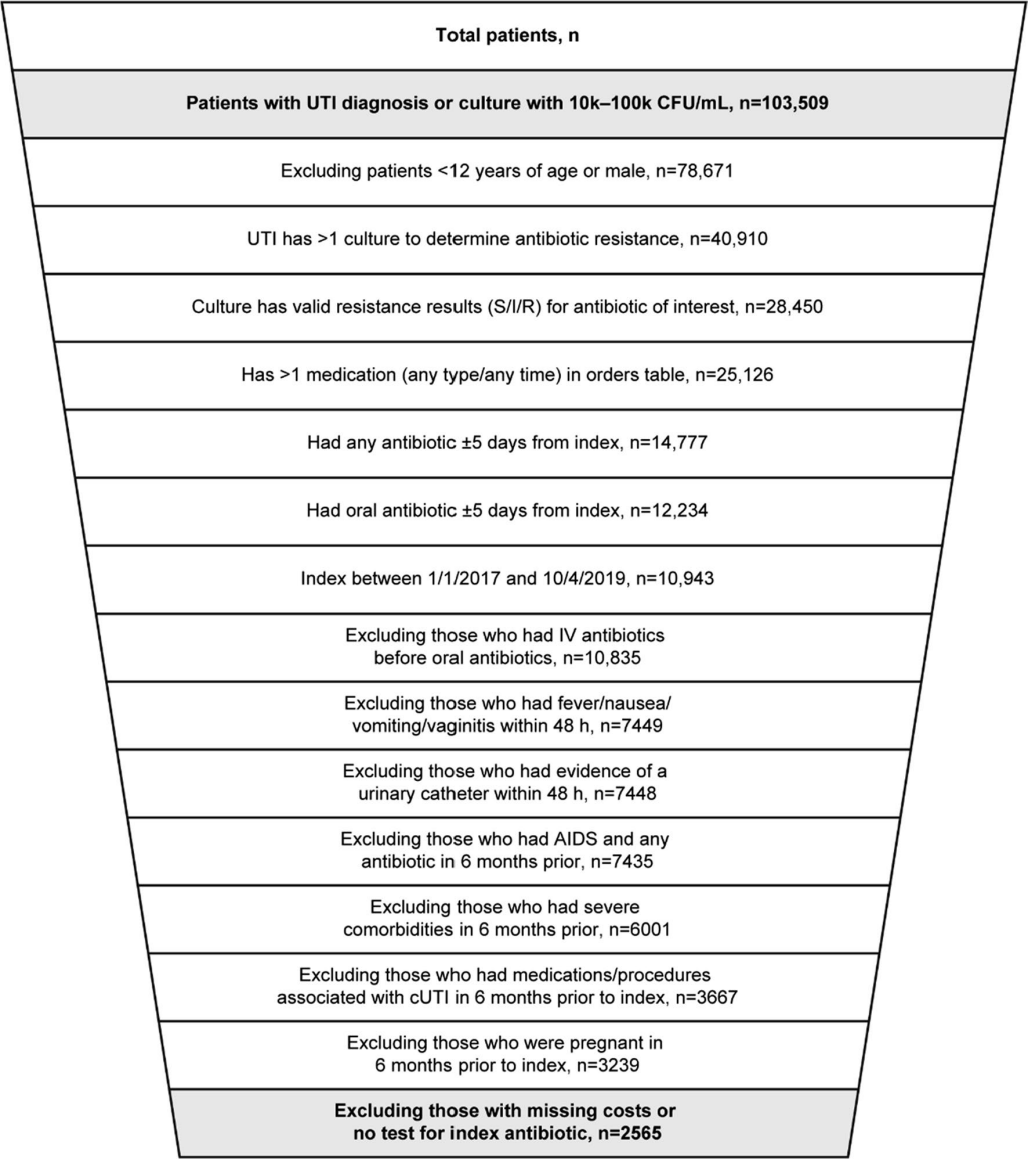
Patients identified with UTI and applied exclusion criteria [18]. *AIDS* acquired immunodeficiency syndrome, *CFU* colony forming units, *cUTI* complicated urinary tract infection, *I* intermediate, *IV* intravenous, *R* resistant, *S* sensitive, *UTI* urinary tract infection

Patients were excluded if they were not prescribed antimicrobial therapy for their uUTI, were pregnant (a complicating comorbidity) at the index uUTI diagnosis, if they had a diagnosis of human immunodeficiency virus/acquired immunodeficiency syndrome (ICD-9: 042, 043, 044; ICD-10: B20-B4) and any antibiotic use from 6 months before to 6 days before the index uUTI date, or had presence of a urinary catheter at index uUTI event or within 48 h of index uUTI. US patients with asymptomatic bacteriuria are not typically prescribed antibiotics, thus patients with an ICD-9/10 diagnosis code for asymptomatic bacteriuria were not included. Patients with acute uncomplicated pyelonephritis were not included; although we understand that acute uncomplicated pyelonephritis and uUTI are treated similarly in certain countries, they are considered distinct conditions which are differentiated by unique ICD diagnosis codes and treated differently in the US. To rule out cases of complicated urinary tract infection (cUTI), eligible patients could not have documentation of fever (temperature ≥ 38.3 °C), nausea, vomiting, flank pain at index uUTI or within 48 h of the index uUTI event, have received intravenous antibiotics as initial therapy (defined as having intravenous antibiotics before the start of oral antibiotic therapy for uUTI within ± 5 days of the index uUTI diagnosis). Patients with urological or renal abnormalities, other structural lesions, or complicating comorbidities (i.e., immunocompromised, complicated diabetes, or pregnancy), or chronic conditions (e.g., malignancy, neutropenia, or diabetes mellitus) that were indicative of cUTI were not included in this analysis. To ensure data completeness, patients with missing laboratory values (i.e., not tested for resistance) or missing utilization measures (i.e., missing inpatient drug order/cost or utilization cost in follow-up period, or missing utilization measure for their index utilization setting) were also excluded from the analysis. Full details of the filtering of the patient dataset have recently been described [18] and are shown in Fig. 2.

### Outcome measures

The goals of the study were to examine: (1) the proportion (n, %) of antibiotics inappropriately or suboptimally prescribed, as identified by laboratory results and allergy history post-index uUTI and according to the definitions below, among patients with a uUTI, and (2) total healthcare costs (all-cause and urinary tract infection [UTI]-related) within 6 months after a uUTI in female patients, stratified by patients with susceptible versus not-susceptible isolates. Total healthcare costs were calculated using HCRU observed in EHR data and multiplied by Medicare fee-for-service rates (for medical services) or the wholesale acquisition cost from ProspectoRx, a real-time online drug pricing and analytics database for pharmaceuticals.

### Exposure and independent variables

Inappropriate treatment was defined as having not been prescribed a recommended antibiotic [12, 15] according to the Infectious Diseases Society of America guidelines [7]. Treatment was considered appropriate if patients were prescribed first-line therapy which consists of fosfomycin, nitrofurantoin, or SXT monotherapy and prescription durations were appropriate for each treatment. Patients with known allergies to first-line antibiotics were prescribed (appropriate) alternate treatments. Allergies were confirmed according to medical record recording of patient allergies. Suboptimal treatment was defined as switching index treatment within 28 days of index due to treatment failure, receiving intravenous antibiotics, or receiving initial or index treatment to which isolates were not-susceptible. Further details for the above treatment definitions are provided in Table 1.

**Table 1:** Definitions of appropriate, inappropriate, and suboptimal antibiotic treatment

Appropriate treatment	Inappropriate treatment	Suboptimal treatment
Patients were prescribed first-line therapy which consists of fosfomycin, nitrofurantoin, or SXT, alone OR Patients with known allergies were prescribed appropriate subsequent line of treatments AND Prescription durations were appropriate to the treatment such as: 5 days for nitrofurantoin 1 day for fosfomycin 3 days for SXT	Patients were prescribed two first-line therapies OR Patients received β lactam agents, fluoroquinolones, or any other second-line or alternate therapy as initial therapy if patients were not allergic or resistant to first-line therapies OR Patients received antibiotics which they showed resistance to OR Patients received antibiotics that they were allergic to, identified by the patient history and drug allergy tables	Patients had to switch from current therapy to another first-line, second-line, or third-line therapy OR Patients received intravenous antibiotics (oral administration is the appropriate route for patients with uUTI)

*SXT* trimethoprim-sulfamethoxazole, *uUTI* uncomplicated urinary tract infection

Antibiotic sensitivity was determined based on urine isolate susceptibility test results. Laboratory reports typically contained a qualitative interpretation, which categorized the results as sensitive, intermediate, and resistant. Sensitive results were categorized as susceptible, whereas resistant and intermediate results were categorized as not-susceptible. For patients with two or more isolates that had different susceptibility results (e.g., susceptible results for one isolate and not-susceptible results for another isolate), the individual was classified as being not-susceptible at the person-level. This was identified by first determining the number of isolates and then using this decision rule to assign a person to a susceptible/not-susceptible status at the person level.

Using the definitions above, the primary exposure variables were a series of 4 indicator variables describing patients that had (1) antibiotic not-susceptible isolate(s) and appropriate prescribing, (2) antibiotic-not-susceptible isolate(s) and inappropriate or suboptimal prescribing, (3) antibiotic-susceptible isolate(s) and inappropriate or suboptimal prescribing, (4) antibiotic-susceptible isolate(s) and appropriate prescribing (reference group).

Other independent variables of interest included demographics (i.e., age, race), health insurance type, and comorbidities (hemiparesis, renal disease, myocardial infarction, chronic pulmonary disease, rheumatic disease, peptic ulcer disease, mild liver disease, moderate or severe liver disease, dementia, peripheral vascular disease, cerebrovascular disease, and congestive heart failure). Comorbidity burden was calculated using the Charlson Comorbidity Index (CCI).

### Statistical analysis

HCRU and costs were compared between patients who had isolates that were antibiotic not-susceptible and those with isolates that were antibiotic susceptible, and between antibiotic appropriateness cohorts using multivariable generalized linear models with a log link and gamma family distribution. All models were adjusted by cohort, baseline CCI, and baseline all-cause HCRU (inpatient, emergency department, outpatient, pharmacy). A *P*-value of < 0.05 was set as the threshold for statistical significance.

When comparing patients with antibiotic-not-susceptible versus antibiotic-susceptible isolates, propensity score matched analysis was performed using a 1:1 propensity score calculation—including age, White race, and having private insurance as covariates. Inpatient visits in the previous 180 days and outpatient clinic or other visits in the previous 180 days were also included as covariates in the analysis of patients with not-susceptible versus susceptible isolates. Costs were adjusted to 2020 US dollars ($) and to minimize the influence of outlier observations, in some specifications, costs were winsorized at the 98th percentile.

Sensitivity analyses were carried out to verify the primary analysis and test the robustness of the findings. Changed parameters included the application of alternative inclusion and exclusion criteria, shortening and extending the follow-up period (30 days and 360 days versus the initial 180-day period), excluding patients with prior infection, and only including patients receiving fluoroquinolone therapy. In the analysis where stricter inclusion criteria were applied, patients were required to have both a diagnosis code and positive urine culture for uUTI inclusion versus having one or the other, while when less strict inclusion criteria from a previous study [19] were applied, patients were only excluded if they met the following criteria: were male; were pregnant; had urinary complications such as genitourinary malignancy or calculus of the kidney; had a chronic indwelling catheter; were using immunosuppression therapy; had uncontrolled diabetes mellitus; or had received intravenous antibiotics as initial therapy prior to the start of oral antibiotic therapy for uUTI within ± 5 days of the index uUTI diagnosis. Patients with prior infection were excluded from one of the sensitivity analyses due to the known additional economic burden caused by infectious diseases.

## Results

Overall, 2565 female patients were included (mean age 43.5 years, 59.5% White). Demographics and clinical characteristics of unmatched and matched patients stratified by isolate susceptibility status are shown in Table 2. The most common comorbidities in the overall population were chronic obstructive pulmonary disease (2.2%), dementia (0.7%), and rheumatic disease (0.6%). In the unmatched patient cohort, patients with susceptible versus not-susceptible isolates were more likely to be younger (mean age 42.8 years versus 44.4 years), White (61.1% versus 58.2%), have private insurance (63.2% versus 58.5%), and have fewer comorbidities (Charlson comorbidity total score 0.053 versus 0.064).

**Table 2:** Patient characteristics before and after propensity score matching, stratified by antibiotic not-susceptible uUTI versus antibiotic susceptible uUTI during first-line therapy

	Unmatched			Matched		
Variable	Susceptible mean (n = 1535)	Not-susceptible mean (n = 1030)	Standard mean difference	Susceptible mean (n = 1009)	Not-susceptible mean (n = 1009)	Standard mean difference
Age, years	42.83	44.42	0.081	43.95	44.12	0.008
*Race, mean proportion of patients*						
White	0.611	0.582	0.060	0.586	0.584	0.004
African American	0.243	0.273	0.068	0.259	0.268	0.020
Asian	0.033	0.034	0.004	0.035	0.035	0
Other race	0.059	0.057	0.006	0.063	0.058	0.021
Unknown race/None/Declined to answer	0.054	0.054	0.001	0.057	0.056	0.009
*Ethnicity, mean proportion of patients*						
Hispanic	0.030	0.030	0.001	0.03	0.031	0.006
Non-Hispanic	0.872	0.876	0.012	0.879	0.873	0.018
Other ethnicity	0.002	0.007	0.073	0.001	0.007	0.095
Unknown ethnicity/None/Declined to answer	0.096	0.087	0.031	0.090	0.089	0.003
*Health insurance, mean proportion of patients*						
Private insurance	0.632	0.585	0.095	0.589	0.595	0.012
Medicare/Medicaid	0.143	0.177	0.091	0.160	0.170	0.029
Other insurance	0.225	0.238	0.031	0.252	0.235	0.039
*Charlson comorbidities, mean numbers*						
Charlson comorbidity total score	0.053	0.064	0.032	0.053	0.054	0.003
COPD	0.021	0.022	0.006	0.021	0.019	0.014
Dementia	0.006	0.009	0.034	0.008	0.005	0.037
Cerebrovascular disease	0.005	0.003	0.027	0.004	0.003	0.017
Rheumatic disease	0.005	0.007	0.021	0.006	0.007	0.012
Congestive heart failure	0.003	0.001	0.039	0.002	0.001	0.026
Mild liver disease	0.003	0.004	0.022	0.001	0.004	0.060
Peripheral vascular disorder	0.003	0.004	0.022	0.003	0.004	0.017
Hemiparesis	0.001	0	0.036	0	0	0
Peptic ulcer	0.001	0	0.036	0.001	0	0.045
Renal disease	0.001	0	0.036	0	0	0
Moderate/Severe liver disease	0	0.001	0.044	0	0.001	0.045
Myocardial infarction	0	0.001	0.044	0	0.001	0.045
*HCRU (180 days pre-index), mean number of events per patient in the 180 days pre-index*						
Inpatient encounters (all cause)	0.004	0.012	0.077	0.001	0.003	0.045
Outpatient encounters (all cause)	1.024	1.066	0.022	1.014	0.995	0.011
Outpatient clinic encounters (all cause)	0.897	0.975	0.046	0.929	0.912	0.011
Outpatient ambulatory surgery encounters (all cause)	0.01	0.014	0.026	0.012	0.009	0.028
Outpatient other encounters (all cause)	0.117	0.078	0.087	0.073	0.074	0.003
Emergency department encounters (all cause)	0.052	0.084	0.103	0.065	0.066	0.003
Drug orders (all cause)	1.742	2.129	0.068	1.847	1.778	0.016
Any antibiotic use	0.098	0.150	0.158	0.097	0.147	0.152

*COPD* chronic obstructive pulmonary disease, *HCRU* healthcare resource use, *uUTI* uncomplicated urinary tract infection

### Antibiotics prescribed

The most commonly prescribed antibiotics were nitrofurantoin (60.8%), SXT (19.4%), and ciprofloxacin (14.6%). Levofloxacin, ciprofloxacin, SXT, nitrofurantoin, and amoxicillin were the most commonly performed susceptibility tests. More than one-third of patients (40.2%) had an isolate which was not-susceptible to ≥ 1 antibiotic indicated for treating patients with uUTI. The proportions of patients allergic to specific antibiotics are shown in Table  3. In total, 133 patients (5.2%) were allergic to at least one antibiotic indicated for uUTI, of whom 77 (57.9%) had susceptible isolates and 56 (42.1%) had not-susceptible isolates.

**Table 3:** Allergy history post-index uUTI

	Susceptible n = 1535		Not-susceptible n = 1030		Difference		Overall n = 2565	
Variable	Mean proportion (%)	n	Mean proportion (%)	n	Difference	Standard mean difference	Mean proportion (%)	n
Any UTI antibiotic	5.0	77	5.44	56	0.42	0.019	5.19	133
Any first-line UTI antibiotic	2.93	45	2.82	29	− 0.12	0.007	2.88	74
Any second-line UTI antibiotic	1.11	17	1.36	14	0.25	0.023	1.21	31
Any third-line UTI antibiotic	1.30	20	2.23	23	0.93	0.071	1.68	43
Amoxicillin	0.33	5	0.87	9	0.55	0.071	0.55	14
Cefaclor	0.58	9	0.49	5	− 0.10	0.014	0.55	14
Cefdinir	0.20	3	0.00	0	− 0.20	0.063	0.12	3
Cefpodoxime	0.00	0	0.00	0	0.00		0.00	0
Ciprofloxacin	0.91	14	1.26	13	0.35	0.034	1.05	27
Fosfomycin	0.00	0	0.00	0	0.00		0.00	0
Levofloxacin	0.46	7	1.07	11	0.61	0.070	0.70	18
Nitrofurantoin	1.04	16	0.58	6	− 0.46	0.051	0.86	22
Ofloxacin	0.00	0	0.00	0	0.00		0.00	0
SXT	1.89	29	2.23	23	0.34	0.024	2.03	52

*SXT* trimethoprim/sulfamethoxazole, *UTI* urinary tract infection, *uUTI* uncomplicated urinary tract infection

**Fig. 3:**
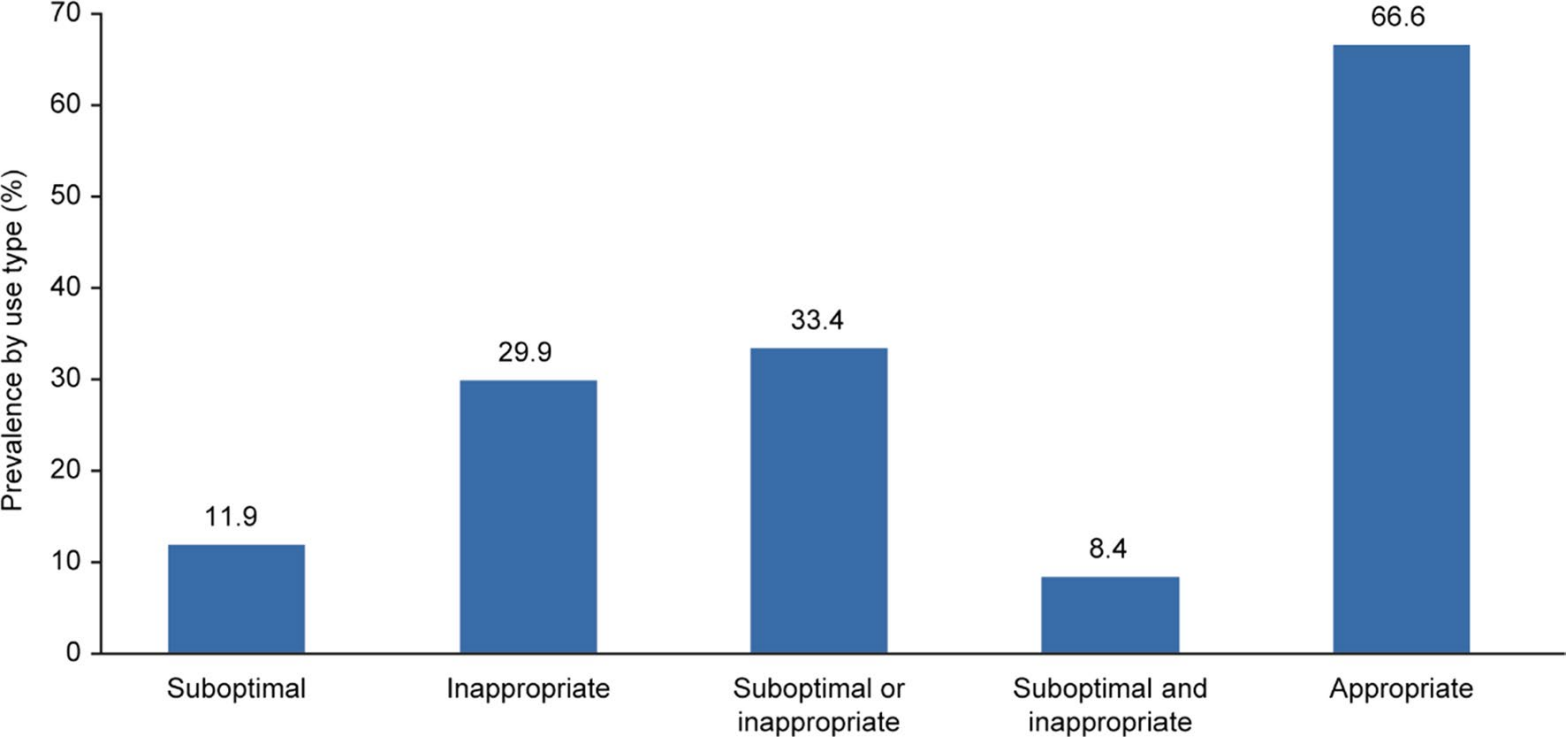
Proportion of antibiotics that are inappropriately or suboptimally prescribed among patients with uUTI. Note: Patients could be classified as receiving both suboptimal or inappropriate care simultaneously. Appropriate = both appropriate and not suboptimal treatment. Suboptimal, n = 306; inappropriate, n = 766; suboptimal or inappropriate, n = 856; suboptimal and inappropriate, n = 215; appropriate, n = 1709. *uUTI* uncomplicated urinary tract infection

The proportion of antibiotics inappropriately and/or suboptimally prescribed among patients with uUTI is shown in Fig. 3. In total, 66.6% (1709/2565) of study-eligible patients received appropriate treatment and 33.4% (856/2565) received suboptimal or inappropriate treatment, with more patients receiving inappropriate treatment (29.9%) than suboptimal treatment (11.9%). Overall, 8.4% of patients received both suboptimal and inappropriate prescriptions.

Inappropriate prescribing was more common for patients with not-susceptible (48.2%, 496/1030) versus susceptible (23.5%, 360/1535) isolates as antibiotic susceptibility was not available to treating physicians at the time prescribing decisions were made.

### Healthcare resource use and costs

**Fig. 4:**
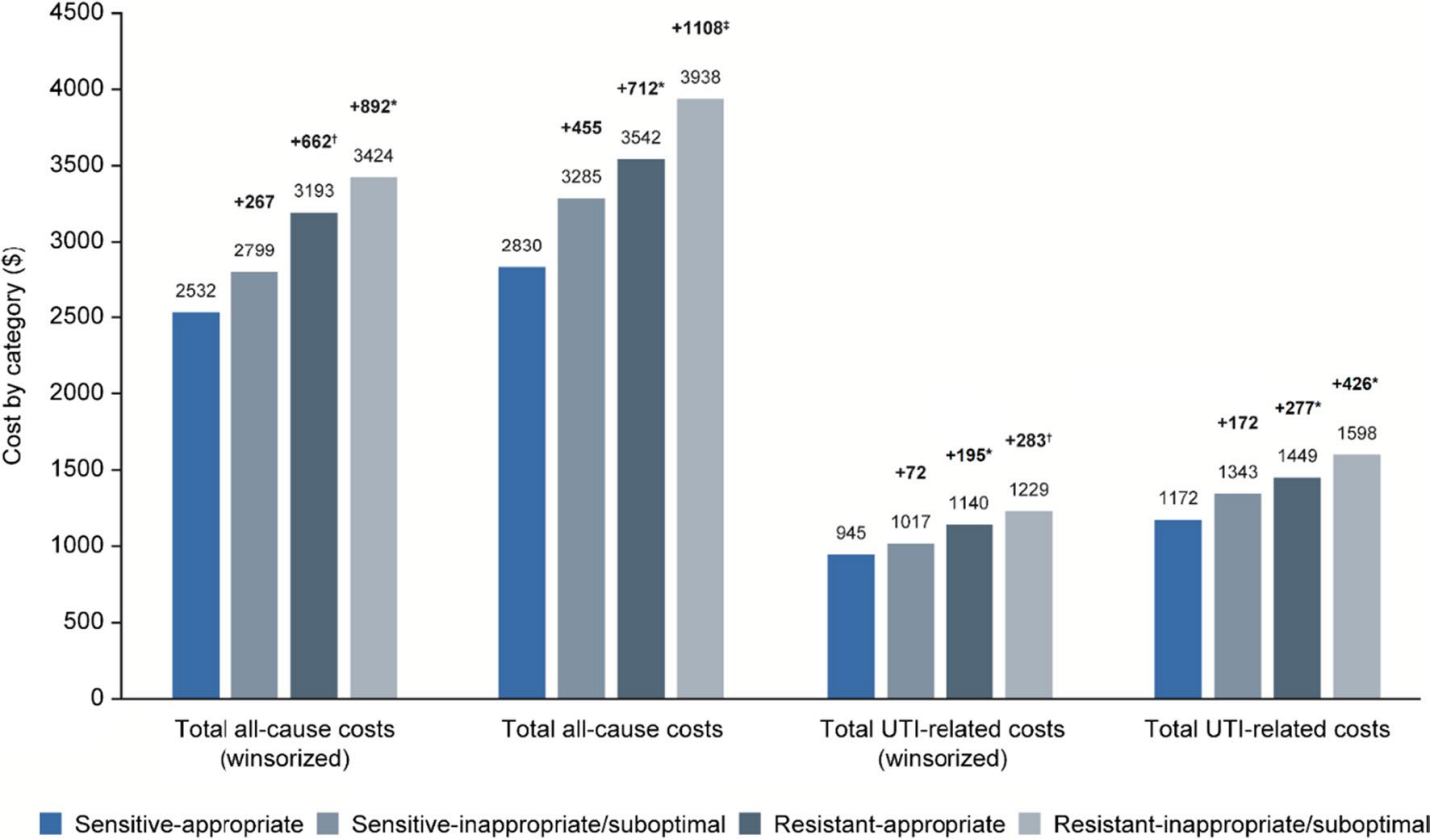
Healthcare costs (UTI-related and all-cause) stratified by susceptible/not-susceptible and appropriate/inappropriate or suboptimal treatment [29]. Difference above susceptible-appropriate and statistical significance (*P < 0.05; ^†^*P* < 0.01; ^‡^*P* < 0.001); susceptible-appropriate, n = 1175; susceptible-inappropriate or suboptimal, n = 360; not-susceptible-appropriate, n = 534; not-susceptible-inappropriate or suboptimal, n = 496. *UTI* urinary tract infection

Inappropriate or suboptimally prescribed patients had higher all-cause costs (+ $427, *P* = 0.050) and significantly higher UTI-related costs (+ $196, *P* = 0.016) compared with appropriately prescribed patients. This was more prominent among patients with antibiotic not-susceptible isolates (Fig. 4). Patients with susceptible isolates that were treated appropriately overall had the lowest all-cause costs ($2532) and UTI-related costs ($945). Patients with susceptible isolates that were inappropriately or suboptimally prescribed had + $267 (*P* = 0.264) greater all-cause costs and + $72 (*P* = 0.426) greater UTI-related costs versus patients with susceptible isolates that were appropriately prescribed. Furthermore, patients with not-susceptible isolates that were appropriately prescribed had + $662 (*P* = 0.003) greater all-cause costs and + $195 (*P* = 0.018) greater UTI-related costs versus patients with susceptible isolates that were appropriately prescribed. Finally, patients with not-susceptible isolates that were inappropriate or suboptimally prescribed had + $892 (*P* < 0.001) greater all-cause costs and + $283 (*P* = 0.001) greater UTI-related costs versus patients with susceptible that were appropriately prescribed.

The sensitivity analysis (Table 4) indicated that the difference in all-cause and UTI-related costs were significantly higher for patients with not-susceptible isolates versus susceptible isolates when stricter exclusion criteria were applied (all-cause: + $798, *P* = 0.014; UTI-related: + $305, *P* = 0.043). No change in cost difference was observed when the exclusion criteria were less strict (all-cause: + $431, *P* = 0.125; UTI-related: + $184, *P* = 0.144). When the follow-up period was shortened to 30 days, there was a decrease in the difference between all-cause costs for patients with not-susceptible isolates versus those with susceptible isolates and a small increase in UTI-related costs, but both were non-significant (all-cause: − $13, *P* = 0.910; UTI-related: + $18, *P* = 0.877). The difference in costs remained non-significant when the follow-up period was extended to 360 days (all-cause: + $412, *P* = 0.285; UTI-related: + $127, *P* = 0.409). Overall, larger albeit non-significant costs were incurred when patients with infectious diseases were excluded (all-cause: + $512, *P* = 0.060; UTI-related: + $207, *P* = 0.085), as was the case when only patients who received fluoroquinolones were included (all-cause: + $606, *P* = 0.316; UTI-related: + $186, *P* = 0.471).

**Table 4:** Sensitivity analyses summary (uUTI patients with susceptible or not-susceptible isolates) [29]

Sensitivity analysis	Sample size	Difference [not-susceptible–susceptible] (*P*-value)									
		Probability of progressing to cUTI		All-cause costs		UTI-related costs		All-cause costs (winsorized)		UTI-related costs (winsorized)	
		Difference	*P*-value	Difference	*P*-value	Difference	*P*-value	Difference	*P*-value	Difference	*P*-value
Baseline	1009	+ 0.060	< 0.001*	+ 430.9	0.125	+ 184.3	0.144	+ 425.7	0.031*	+ 156.8	0.034*
#1 (strict exclusion)	661	+ 0.039	0.009*	+ 797.7	0.014*	+ 304.6	0.043*	+ 653.5	0.002*	+ 217.5	0.028*
#2 (loose exclusion)	1009	+ 0.060	< 0.001*	+ 430.9	0.125	+ 184.3	0.144	+ 425.7	0.031*	+ 156.8	0.034*
#3 (30-day follow-up)	1200	+ 0.057	< 0.001*	− 12.85	0.910	+ 17.57	0.877	+ 36.00	0.614	+ 22.65	0.555
#4 (360-day follow-up)	834	+ 0.058	< 0.001*	+ 412.2	0.285	+ 126.7	0.409	+ 301.9	0.256	+ 65.25	0.478
#5 (infectious disease exclusion)	955	+ 0.058	< 0.001*	+ 511.7	0.060	+ 206.5	0.085	+ 471.7	0.012*	+ 73.15	0.139
#6 (FQ-only)	166	+ 0.036	0.387	+ 605.8	0.316	+ 186.3	0.471	+ 484.9	0.307	+ 196.0	0.369

Shafrin J. Progression of an uncomplicated urinary tract infection among female patients with susceptible and non-susceptible urine isolates: findings from an integrated delivery network. [Oral] presented at IDWeek; September 29–October 3, 2021; Virtual event. https://idweek.org [29]

^*^Statistically significant (*P*-value < 0.05)

*cUTI* complicated urinary tract infection, *FQ* fluoroquinolones, *UTI* urinary tract infection, *uUTI* uncomplicated urinary tract infection

## Discussion

In this study, one-third (33.4%; 856/2565) of patients with uUTI were prescribed antibiotics inappropriately or suboptimally. Of these, most patients were receiving inappropriate prescriptions (29.9% of all patients); however, this is likely due to patients receiving a prescription before their susceptibility test results were available. More than one-third of the sample (40.2%) were not-susceptible to ≥ 1 antibiotic indicated for treating patients with uUTI. Inappropriate prescribing was more common for patients with not-susceptible versus those with susceptible isolates, and 8.4% of patients received both suboptimal and inappropriate prescriptions. Patients prescribed inappropriately or suboptimally had both higher winsorized and non-winsorized healthcare costs (UTI-related and all-cause) compared with appropriately prescribed patients. Furthermore, healthcare costs were also higher in patients with antibiotic not-susceptible isolates.

The treatment pattern results we report align with those of other studies in patients with uUTI, although it should be noted that direct comparisons cannot be made between studies due to variation in patient population size and the number and variety of healthcare centers examined, particularly in larger studies [6, 15, 16, 20–24] versus our study. Several retrospective studies have also found that the prevalence of inappropriate and/or suboptimal antibiotic prescribing is high in the treatment of uUTI, which may have implications for patient health outcomes [6, 15, 16, 20–24]. In a retrospective cohort study of outpatient and emergency department visits within a US commercial insurance database, inappropriate antibiotics were prescribed for uUTI in approximately 50% of patients [15]. Moreover, wide variations were observed in the duration of antibiotic treatment, with > 75% of prescriptions being for non-recommended durations [15]. In another retrospective cohort study examining the first-line use of antibiotics in female patients with uUTI in the US, 88.7% had inappropriate or suboptimal antibiotic use [16]. Inappropriate drug class assignment occurred in 53.4% and inappropriate therapy duration occurred in 46.6% of patients [16]. Other studies in elderly and pediatric patients have also reported similarly high rates (65% to 70%) of suboptimal/inappropriate antibiotic treatment of patients [20–22].

Some studies have linked suboptimal use of antibiotics in patients with UTIs to poor clinical outcomes [22] or increased costs [20, 24]. In a national cohort study, Appaneal et al. [22] showed that, compared with optimal antibiotic treatment, suboptimal treatment was associated with a 6% increased risk of a composite measure of poor clinical outcome. It was suggested that this was driven by an 94% increased risk of *Clostridioides difficile* infection [22]. In addition, Al-Sayyed et al. [21] showed that inappropriate diagnosis and treatment of uUTI leads to unnecessary costs and estimated that the cost of antibiotic treatment in patients who were inappropriately diagnosed was $10,755.87 [20]. Separately, Kahan et al. [22] demonstrated that suboptimal adherence to treatment guidelines for uUTI led to a waste of healthcare resources. The expected cost of therapy was exceeded in approximately 70% of cases [24].

The present results align with prior studies that indicated antimicrobial resistance was associated with higher treatment costs [13, 14, 25, 26]. Additional need for urine culture and susceptibility testing due to antibiotic resistance increases the use of healthcare resources and subsequently costs [13]. In a matched cohort study including adults with UTI admitted to a tertiary care hospital in Barcelona, Spain, Esteve-Palau et al. [26] reported increased costs associated with extended spectrum beta-lactamase (ESBL)-producing infections. Compared with non-ESBL infections, total pharmacy costs and antibiotic costs, as well as costs associated with outpatient parenteral antibiotic therapy, were higher for patients harboring ESBL-producing infections [26]. Moreover, in a systematic review of the literature, Merritt et al. found that patients with community-acquired UTIs caused by not-susceptible strains of *Escherichia coli (E. coli)* were associated with additional outpatient medical care, and increased overall costs of care compared to patients with antibiotic susceptible strains [14].

Several limitations of the present study should be noted. This analysis was conducted in a single integrated delivery network and the results of this study should not be extrapolated to a regional level. Despite this limitation, the Mid-Atlantic area where the study database is located has a diverse population both in terms of population density (i.e., urban, suburban, and rural areas), as well as its racial and socioeconomic composition. This population diversity is important. A recent study has demonstrated differences in antibiotic prescribing for uUTI in rural versus urban regions, with women in rural areas shown to be more likely to receive prescriptions with inappropriately long durations than women in urban regions [23]. It is important to note that the present study does not differentiate degree of resistance for each patient isolate. This is relevant as HCRU for a patient with a multi-drug resistant isolate may differ from that of a patient with an isolate that is resistant to one drug or drug class. Additionally, the diagnosis of uUTI may be imperfect within the database. For example, urine cultures may not be performed randomly, which may lead to biased sampling. In addition, the requirement of complete data for eligible patients (laboratory values, utilization measures, and costs) could have potentially introduced selection bias in that the patients with complete measures may be different than those without complete measures. The relatively strict eligibility criteria may also have led to a potential selection bias towards more severe cases or recurrent uUTI that may be associated with a higher likelihood of antibiotic use, antibiotic resistance, and associated HCRU and costs than the general uUTI population (e.g., those treated empirically). Additionally, patients with uUTI who were not prescribed antibiotic therapy or received alternative non-antibiotic therapies were not eligible, although this only represents a subset of all patients with uUTI. This was because it was not possible to discern if an antibiotic prescription was deemed unnecessary based on these data, such that this study focused on patients with confirmed infection, confirmed resistance, and confirmed treatment, and our findings may not be generalizable to all patients with uUTI. Also, the use of ICD-9 or ICD-10 codes could overestimate or underestimate the diagnosis of uUTI in the database since some of the codes are dependent on the hospital coder rather than the clinician. The accuracy of identifying a uUTI diagnosis is only as accurate as the detail physicians have supplied in the EHR. Medical records could also contain misclassification between uncomplicated and complicated uUTI if the proper symptoms are not noted in the EHR. Other specifics of treatment or follow-up were not always captured in the EHR and therefore cannot be commented upon in this analysis. Medications received over-the-counter—such as non-steroidal anti-inflammatory agents or phenazopyridine for urinary pain relief—would not be included in the EHR data if purchased by patients outside the health system pharmacy. In addition, EHR record data did not record subsequent data in patient cases where the individual moved or changed their regular provider. However, as uUTI episodes are relatively short, this is a modest concern.

uUTIs that are community-acquired are typically treated in outpatient settings with antibiotic prescriptions based on treatment guidelines and on patient symptoms [14]. Empiric treatment of uUTIs, i.e., without specific knowledge of the pathogen or antibiotic susceptibility, is sometimes necessary as susceptibility testing takes time and results may not be available at the initial consultation. However, by using an empiric approach, patients might be prescribed an inappropriate or suboptimal therapy, which may lead to a higher probability of treatment failure and subsequent antibiotic not-susceptible infections [27]. As the incidence of antibiotic resistance has significantly increased in the US among community-acquired uUTIs [6, 28], it is critical to understand regional resistance rates through local community surveillance to inform and improve empiric prescribing. More rapid diagnostic tests are needed in order to optimize prescribing accuracy and avoid manifestation of painful symptoms. It should be noted that while the inclusion of both urine culture and antibiotic susceptibility testing data is a strength of this study, the findings may not apply to patients who do not have a routine culture. In the absence of timely susceptibility testing, point of care tests to detect resistance phenotypes among the most prevalent uUTI isolates, e.g., *E. coli*, will allow more appropriate empiric prescribing. Additionally, empiric treatment could be enhanced by generating dynamic, real-time antibiograms by mining the data from a hospital’s EHR records of resistance patterns to regionalize and localize treatment recommendations.

## Conclusions

Suboptimal or inappropriate antibiotic prescribing for uUTI is common and is associated with higher healthcare costs than appropriate treatment, particularly among patients with antibiotic-not-susceptible isolates versus those with antibiotic-susceptible isolates. These findings underline the need to improve prescribing accuracy by better understanding of regional resistance rates and developing more rapid diagnostic tests.

## Data Availability

The authors confirm that the data supporting the findings of this study are available within the article and its supplementary materials.

## Abbreviations

CCI: Charlson comorbidity index
CFU: Colony forming unit
cUTI: Complicated urinary tract infection
*E. coli*: *Escherichia coli*
EHR: Electronic health record
ESBL: Extended spectrum beta-lactamase
HCRU: Healthcare resource use
ICD-9: International Classification of Disease, Ninth Revision
ICD-10: International Classification of Disease, Tenth Revision
SXT: Trimethoprim/sulfamethoxazole
uUTI: Uncomplicated urinary tract infection
US: United States
UTI: Urinary tract infection
